# A114 UNIFIED MAGNIFYING ENDOSCOPIC CLASSIFICATION (UMEC) FOR GASTROINTESTINAL LESIONS: A NORTH AMERICAN EDUCATION STUDY

**DOI:** 10.1093/jcag/gwac036.114

**Published:** 2023-03-07

**Authors:** M R A Fujiyoshi, Y Fujiyoshi, N Gimpaya, R Bechara, T Jeyalingam, N C Calo, N Forbes, R Khan, M Atalla, A Toshimori, Y Shimamura, M Tanabe, J Mosko, H Inoue, S Grover

**Affiliations:** 1 Division of Gastroenterology, St. Michael's Hospital, University of Toronto, Toronto, Canada; 2 Digestive Diseases Center, Showa University Koto Toyosu Hospital, Tokyo, Japan; 3 Division of Gastroenterology, Kingston General and Hotel Dieu Hospital, Queen's University, Kingston; 4 Division of Gastroenterology, University Health Network, University of Toronto, Toronto; 5 Division of Gastroenterology, University of Ottawa, Ottawa; 6 Division of Gastroenterology, University of Calgary, Calgary, Canada

## Abstract

**Background:**

Magnification endoscopy and magnification narrow-band imaging are image enhanced endoscopy technologies that may allow for the diagnosis of advanced neoplasia in the GI tract on the basis of imaging characteristics. Recently, the Unified Magnifying Endoscopic Classification (UMEC) has been developed, which unified the criteria for the esophagus, stomach, and colon. UMEC divides optical diagnosis into one of the three categories: non-neoplastic, intramucosal neoplasia, and deep submucosal invasive cancer.

**Purpose:**

The objective of this study is to educate North American endoscopists on the use of the UMEC schema, and to ascertain performance of the UMEC framework among North American endoscopists.

**Method:**

Using UMEC, five North American endoscopists (>1000 procedures) without prior training in magnifying endoscopy independently diagnosed previously collected endoscopic image set of the esophagus, stomach, and colon. The endoscopists were trained on the use of UMEC via an eleven-minute training video with exemplars of each element of UMEC from esophagus, stomach, and colon. All endoscopists were blinded to white-light and non-magnifying NBI findings as well as histopathological diagnosis. The diagnostic performance of UMEC was assessed while using the gold standard histopathology as a reference.

**Result(s):**

A total of 299 gastrointestinal lesions (77 esophagus, 92 stomach, and 130 colon) were assessed using UMEC. For esophageal squamous cell carcinoma, the sensitivity, specificity, and accuracy for all 5 endoscopists ranged from 65.2% (95% CI: 50.9–77.9) to 87.0% (95% CI: 75.3–94.6), 77.4% (95% CI: 60.9–89.6) to 96.8% (95% CI: 86.8–99.8), and 75.3% to 87.0%, respectively. For gastric adenocarcinoma, the sensitivity, specificity, and accuracy for all 5 endoscopists ranged from 94.9% (95% CI: 85.0–99.1) to 100%, 52.9% (95% CI: 39.4–66.2) to 92.2% (95% CI: 82.7–97.5), and 73.3% to 93.3%, respectively. For colorectal adenocarcinoma, the sensitivity, specificity, and accuracy for all 5 endoscopists ranged from 76.2% (95% CI: 62.0–87.3) to 83.3% (95% CI: 70.3–92.5), 89.7% (95% CI: 82.1–94.9) to 97.7% (95% CI: 93.1–99.6), and 86.8% to 90.7%, respectively.

**Image:**

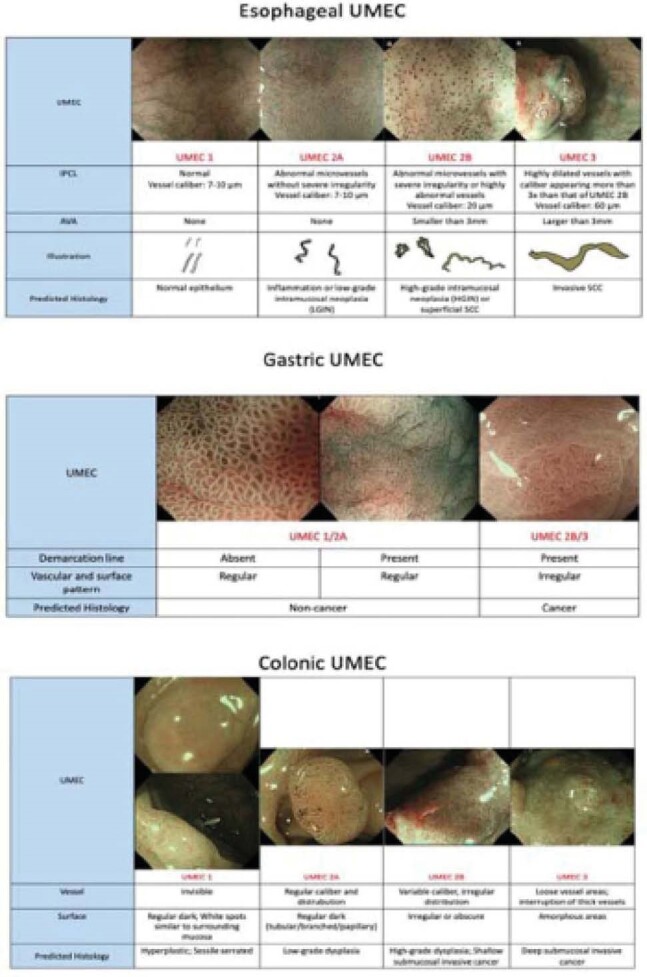

**Conclusion(s):**

UMEC is a simple and practical classification that can be used to introduce and educate endoscopists to magnification narrow-band imaging and optical diagnosis.

**Please acknowledge all funding agencies by checking the applicable boxes below:**

CAG

**Disclosure of Interest:**

M. R. A. Fujiyoshi Grant / Research support from: 2022 CAG/AbbVie Education Research Grant, Y. Fujiyoshi: None Declared, N. Gimpaya: None Declared, R. Bechara: None Declared, T. Jeyalingam: None Declared, N. Calo: None Declared, N. Forbes: None Declared, R. Khan: None Declared, M. Atalla: None Declared, A. Toshimori: None Declared, Y. Shimamura: None Declared, M. Tanabe: None Declared, J. Mosko: None Declared, H. Inoue: None Declared, S. Grover: None Declared

